# Precise predictions for double-Higgs production via vector-boson fusion

**DOI:** 10.1140/epjc/s10052-020-08610-7

**Published:** 2020-11-09

**Authors:** Frédéric A. Dreyer, Alexander Karlberg, Jean-Nicolas Lang, Mathieu Pellen

**Affiliations:** 1 Clarendon Laboratory, Rudolf Peierls Centre for Theoretical Physics, University of Oxford, Parks Road, Oxford, OX1 3PU UK; 2 Physik-Institut, Universität Zürich, 8057 Zurich, Switzerland; 3 Cavendish Laboratory, University of Cambridge, 19 JJ Thomson Avenue, Cambridge, CB3 0HE UK

## Abstract

Theoretical predictions with next-to-next-to-leading order (NNLO) QCD accuracy combined with the next-to-leading order (NLO) electroweak (EW) corrections are presented for differential observables of the double-Higgs production process via vector-boson fusion. While the QCD corrections were previously known, the EW ones are computed here for the first time. The numerical results are obtained for a realistic experimental set-up at the LHC and are presented in the form of fiducial cross sections and differential distributions. Within this setup we find that the VBF approximation employed in the NNLO QCD correction is accurate at the sub-percent level. We find that the NLO EW corrections within the fiducial volume are $-\,6.1\%$, making them of almost the same order as the NLO QCD corrections. In some kinematic regions they can grow as large as $-\,30\%$ making them the dominant radiative corrections. When the EW corrections are combined with the NNLO QCD corrections we find a total correction of $-\,14.8\%$. The results presented here thus comprise the state-of-the-art theoretical predicition for the double-Higgs production via vector-boson fusion, which will be of value to the high-luminosity programme at the LHC.

## Introduction

The discovery of the Higgs boson at the Large Hadron Collider (LHC) [1, 2] opened the door to a new era in high-energy physics, dominated by the quest for precision, and which will culminate in the next decade with the ambitious high-luminosity programme at the LHC [3]. The determination of all the fundamental parameters of the Higgs sector will be at the heart of this programme. Beyond the mass and the width of the Higgs boson, its interactions with the other particles of the Standard Model (SM) will be precisely scrutinised. One of the most far-reaching tasks will be the determination of the Higgs self-couplings. These are fundamental parameters of the SM Lagrangian that determine the shape of the Higgs potential. The investigation of these properties will rely notably on the investigation of processes involving a pair of Higgs bosons in the final state [4–16].

The production of two Higgs bosons via vector-boson fusion (VBF) i.e. $\text {p} \text {p} \rightarrow \text {H} \text {H} \text {j} \text {j}$ is a particularly important process for the determination of the triple-Higgs coupling [17]. While gluon fusion is the dominating double-Higgs production mode [18–22], the VBF channel offers unique opportunities for measurements of Higgs pair production. In VBF, the Higgs bosons are produced at leading order from heavy gauge bosons that are themselves radiated off two quark lines. These two quarks offer a useful handle as tagging jets for its experimental measurement, providing a promising channel for studies of the trilinear and quartic Higgs couplings at the LHC [17, 23].

While single-Higgs production via VBF has been measured [24], its double-Higgs counterpart is not yet accessible with the current experimental data set. The reason is that the cross section at $14\,\,\text {TeV}$ is of the order of$1 {\,\text {fb}}$ as it is a purely electroweak (EW) process and requires very exclusive event selections in order to single it out from its background. Nonetheless, this process is already used to look for new physics [25]. The high-luminosity programme of the LHC is aiming at collecting about$3000{\,\text {fb}} ^{-1}$ in the next two decades. This should allow the observation of the double-Higgs production via VBF. To that end, precise and reliable theoretical predictions are required.

In this article we provide for the first time next-to-leading order (NLO) EW corrections and combine them with the existing next-to-next-to-leading order (NNLO) QCD corrections. Both types of corrections are very different in magnitudes and shapes as they account for different physical effects. The QCD ones are usually larger and their missing higher-order terms can be well estimated by a variation of the renormalisation and factorisation scales. In Refs. [26, 27], it has been shown that only NNLO QCD corrections provide reliable predictions at the differential level. Recently, the $\mathrm{N}^3\mathrm{LO}$ corrections to the inclusive cross section have also been computed, and were shown to be at the few permille level [28]. The EW corrections on the other hand are typically rather suppressed and appear in the high-energy tail of differential distributions where the effect of Sudakov logarithms become large.

The present article is organised as follow: in the first part, the details of the computation are provided. In particular the numerical inputs and the event selection are explained. In the second part, the results are given in the form of cross sections and differential distributions. Finally, the conclusion contains a short summary of the article as well as concluding remarks.

## Details of the computation

### Description of the process

The process of interest is the double-Higgs production via VBF, which can be expressed as

1 $$\begin{aligned} \text {p} \text {p} \rightarrow \text {H} \text {H} \text {j} \text {j}. \end{aligned}$$

**Fig. 1:**
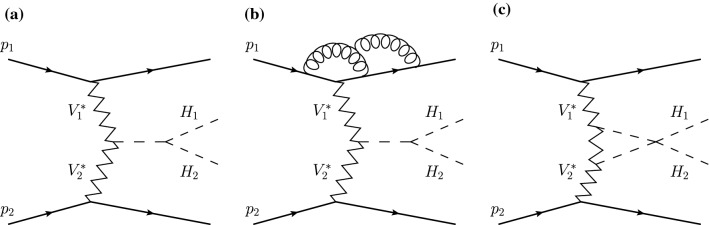
Examples of Feynman diagrams contributing to the VBF Higgs pair production process at LO (**a**), NNLO QCD (**b**) and NLO EW (**c**)

It is a purely EW process which is defined at tree level at the order $\mathcal {O}\left( \alpha ^4 \right)$. At this order, it contains the VBF topology which is defined as two quark lines that radiate heavy gauge bosons that fuse to give rise to two Higgs bosons, as shown in Fig. 1a. Alternatively, the two Higgs bosons can be radiated from a heavy gauge boson decaying subsequently into two quarks. The latter topology is usually referred to as Higgs Strahlung. While these two topologies are intimately related, they entail rather different physical effects.

It has lead, on the experimental side, to consider the two processes separately by imposing rather stringent cuts on the invariant mass and the rapidity difference of the two tagging jets. This has motivated the use of the VBF approximation for theoretical predictions, which neglects s-channel contributions as well as t/u interferences in a gauge-invariant way. Additionally the two quark lines are not allowed to exchange virtual or real gluons. One advantage of this approach is that it greatly simplifies the computations. In particular, it has allowed the computation of QCD corrections up to N$^3$LO of single and double Higgs production [26, 28–34]. The differential cross section of the di-Higgs process was also computed at NLO with matching to parton showers in this approximation [35].

In the present article, we have used the full computations at LO, NLO QCD, and NLO EW while we have utilised the VBF approximation at NNLO. In order to justify their combination, we have used a rather exclusive experimental set-up where the VBF approximation holds at the per-mille level. In addition, we have added a correction factor to the NNLO corrections to account for the differences between the full and the VBF computation. More precisely we have checked that the difference between the full computation and the VBF-approximate one is identical at LO and NLO QCD in all differential distributions. Recently, extensive studies investigating the quality of such approximation at NLO in QCD for vector-boson scattering (VBS) [36] and the EW production of a Higgs boson in association with three jets [37] have been performed. We have refrained from presenting similar results for the process at hand as it is very close to processes mentioned above. Instead we have displayed the correction factor used in all differential distributions which indicates the difference between the full and the approximate computation.

- QCD corrections
  In the full computation at NLO, the real corrections consist in all the contributions of the type $\text {p} \text {p} \rightarrow \text {H} \text {H} \text {j} \text {j} \text {j}$ at order $\mathcal {O}\left( \alpha _\text {s} \alpha ^4 \right)$. The virtual corrections entail all the possible gluon insertions on a given quark line, with gluon exchanges between the two quark lines vanishing for colour reasons. At NNLO, for which an example diagram is shown in Fig. 1b, heavy-quark loop-induced diagrams are neglected, as well as *t*-/*u*-channel interferences, single-quark line contributions and loop induced interferences between VBF and gluon-fusion production. These contributions have been shown to be suppressed to less than a percent in the single-Higgs case [38–40], but are not known for Higgs-pair production. Although they could be sizeable there is no apriori reason to expect them to be enhanced in di-Higgs production. A dedicated future study is however necessary to confirm whether or not these effects can be neglected. The non-factorisable diagrams involving the exchange of two gluons between the quark lines, have recently been estimated in Ref. [27]. Unlike their counterparts in single Higgs VBF production [41], they have been shown to be of the same order as the corrections arising from gluon exchanges limited to one quark line, the so-called factorisable corrections shown in Fig. 1b. In this work we therefore also provide an estimate of the non-factorisable corrections, but will show them separately from the factorisable corrections. Unless explicitly specified, when referring to NNLO QCD corrections, we will always mean the factorisable ones. We compute the factorisable NNLO QCD corrections using the projection-to-Born method as detailed in Ref. [26].
- EW corrections
  For the EW corrections the real radiations are made of the $\text {p} \text {p} \rightarrow \text {j} \text {j} \text {H} \text {H} \gamma$ channels at order $\mathcal {O}\left( \alpha ^5 \right)$. At the same order, the virtual corrections are obtained by inserting EW particles anywhere possible in the tree-level topologies, an example of which is shown in Fig. 1c. Note that at the order $\mathcal {O}\left( \alpha ^5 \right)$, photon-induced contributions also arise. These have been neglected in the present work as these have been shown to be rather small for similar processes [38, 42, 43].^1^ Note that EW corrections to single-Higgs production have been computed for the first time in Refs. [38, 46] and are available in HAWK [44]. Later they have also been obtained in VBFNLO [47, 48].

As mentioned previously, all LO and NLO predictions are based on the full computation, i.e. without employing the VBF approximation. These have been obtained from the Monte Carlo MoCaNLO, which has already been used for a variety of processes and in particular VBS ones [42, 43, 49] at NLO EW and NLO QCD. The matrix elements are provided by Recola [50–52] which internally uses the Collier library [53, 54] to evaluate tensor integrals.

On the other hand, the NNLO QCD corrections have been obtained from proVBFHH v1.1.0 [26, 28] which uses the projection-to-Born method [32] to compute the fully differential NNLO corrections in the VBF approximation. In order to correct for the mismatch between this computation and the full computation used for the LO and NLO computations, we compute a differential correction factor

2 $$\begin{aligned} K_{\mathrm {full/VBF}} = \frac{d\sigma ^\mathrm{full}_\mathrm{LO}}{d\sigma ^\mathrm{VBF}_\mathrm{LO}} \end{aligned}$$

and obtain the NNLO cross section provided below in the following way

3 $$\begin{aligned} \sigma _\mathrm{NNLO\; QCD} = \sigma ^\mathrm{full}_\mathrm{LO} + \delta ^\mathrm{full}_\mathrm{NLO\; QCD} + K_{\mathrm {full/VBF}}\delta ^\mathrm{VBF}_\mathrm{NNLO\; QCD}, \end{aligned}$$

where the *full* quantities refer to the computations with no approximations and the *VBF* one to the relative NNLO corrections in the VBF approximation. At the differential level, the NNLO predictions are obtained in the same way. We have checked numerically that if one were to obtain $K_{\mathrm {full/VBF}}$ using instead the NLO cross section, the results do not change within statistical uncertainties. This is only true under the very stringent cuts that we will introduce below.

The non-factorisable NNLO QCD corrections, $\delta ^\mathrm{NF}_\mathrm{NNLO\; QCD}$, have also been obtained using proVBFHH v1.1.0 as computed in Ref. [27]. Since they are very different in nature from the factorisable corrections, we do not correct them by $K_{\mathrm {full/VBF}}$, and show them separately from all other results.

Finally, to combine the NNLO QCD prediction and the NLO EW ones, we have followed the Higgs cross section working group recommendation [55] for VBF which reads

4 $$\begin{aligned} \sigma _\mathrm{NNLO \; QCD \times NLO \; EW} = \sigma _\mathrm{NNLO \; QCD} \left( 1 + \frac{\delta ^\mathrm{full}_\mathrm{NLO \; EW}}{\sigma ^\mathrm{full}_\mathrm{LO}} \right) , \end{aligned}$$

with $\sigma ^\mathrm{full}_\mathrm{NLO \; EW} = \sigma ^\mathrm{full}_\mathrm{LO} + \delta ^\mathrm{full}_\mathrm{NLO \; EW}$. The same formula has been applied differentially. With such a prescription we provide thus best predictions at fixed order in the SM for double-Higgs production in VBF. We stress that we do not include $\delta ^\mathrm{NF}_\mathrm{NNLO\; QCD}$ in this quantity, but rather quote it separately.

### Numerical inputs

Our predictions are obtained for proton-proton collisions at the LHC running with a centre-of-mass energy of $\sqrt{s} = 14 \,\,\text {TeV}$. The 5-flavour scheme is used throughout the computation i.e. the bottom quarks are considered massless. Bottom quarks are also included in the jet definition. For the parton distribution functions (PDF) and $\alpha _s$, the NNPDF31_nnlo_as_0118_luxqed set [45] has been used for all computations i.e. at LO, NLO, and NNLO as implemented in LHAPDF6 [56]. The choice of the central renormalisation and factorisation scale is the same as the one used in Ref. [26] and reads

5 $$\begin{aligned} \mu = \sqrt{\frac{M_\text {H}}{2} \sqrt{\left( \frac{ M_\text {H}}{2} \right) ^2 + p_{\text {T},\text {H} \text {H}} ^2}} , \end{aligned}$$

with $M_\text {H}$ the mass of the Higgs boson and $p_{\text {T},\text {H} \text {H}}$ the transverse momentum of the di-Higgs system. In order to estimate the impact of missing higher-order QCD corrections, we perform a 3-point scale variation defined by $\mu _\mathrm{ren} = \mu _\mathrm{fac} = \{1/2,1,2\}\times \mu$. Note that this method is applied for the LO predictions and the factorisable QCD corrections only. For the EW corrections, such a method does not provide a good estimate of missing higher-order and thus NLO EW corrections are simply applied as an overall factor.

The masses and widths used for the numerical simulations read

6 $$\begin{aligned} \begin{aligned} m_\text {t}&= 173.21\,\,\text {GeV},&\quad \quad \quad M_\text {H}&= 125.0\,\,\text {GeV}, \\ M_\text {Z} ^\text {OS}&= 91.1876\,\,\text {GeV},&\quad \quad \quad \Gamma _\text {Z} ^\text {OS}&= 2.4952\,\,\text {GeV}, \\ M_\text {W} ^\text {OS}&= 80.385\,\,\text {GeV},&\Gamma _\text {W} ^\text {OS}&= 2.085\,\,\text {GeV}. \end{aligned} \end{aligned}$$

The mass of the bottom quark is taken to be zero in accordance with the 5-flavour scheme. The width of the top quark is also taken to be zero as no top quarks are produced resonantly. The value taken for the Higgs-boson mass is taken from the report of the Higgs cross section working group [55]. Note that the width has been taken to zero as the Higgs bosons are on-shell external states. They can nonetheless appear as internal propagator in the splitting $\text {H} ^* \rightarrow \text {H} \text {H}$. As the invariant mass of the off-shell Higgs boson tends to be far from the on-shell mass and since the value of the width, $\Gamma _\text {H} = 4.07 \times 10^{-3}\,\,\text {GeV}$, is small, setting the width to zero has no numerical impact.^2^ The pole masses and widths used for the simulations are obtained from the measured on-shell (OS) values [57] for the W and Z bosons according to

7 $$\begin{aligned} M_V= & {} \frac{M_{V}^\text {OS}}{\sqrt{1+(\Gamma _{V}^\text {OS}/M_{V}^\text {OS})^2}},\nonumber \\ \Gamma _V= & {} \frac{\Gamma _{V}^\text {OS}}{\sqrt{1+(\Gamma _{V}^\text {OS}/M_{V}^\text {OS})^2}}, \end{aligned}$$

with $$V=\text {W}, \text {Z} $$.

The $G_\mu$ scheme [58] is used for all computations and is translated to $\alpha$ via

8 $$\begin{aligned} \alpha= & {} \frac{\sqrt{2}}{\pi } G_\mu M_\text {W} ^2 \left( 1 - \frac{M_\text {W} ^2}{M_\text {Z} ^2} \right) \qquad \text {and}\nonumber \\ G_\mu= & {} 1.16637\times 10^{-5}\,\,\text {GeV}. \end{aligned}$$

In all the LO and NLO computations, the intermediate W/Z-boson resonances are treated in the complex-mass scheme [59–61] to ensure gauge independence of all amplitudes.

### Event selection

The experimental cuts are rather generic and typically intend to single out VBF EW contributions from their QCD background. To that end, high invariant-mass and large rapidity difference between the two tagging jets are required. The tagging jets are defined by requiring that each jet fulfils the following condition:

9 $$\begin{aligned} p_{\text {T},\text {j}} > 25\,\,\text {GeV} \quad \mathrm{and} \quad |y_{\text {j}}| < 4.5 . \end{aligned}$$

The jets are clustered using the anti-$k_t$ algorithm [62] with $R=0.4$, using FastJet v3.3.0 [63] in the case of proVBFHH. The two hardest jets in the transverse momentum fulfilling these requirements are required to obey the VBF-selection cuts which read

10 $$\begin{aligned} m_{\text {j} _1 \text {j} _2}> 600\,\,\text {GeV} \quad \mathrm{and} \quad |y_{\text {j} _1} - y_{\text {j} _2}| > 4.5. \end{aligned}$$

Note that we do not apply a cut ensuring that both tagging jets are in opposite hemispheres as in Ref. [26]. Also, the event selection is completely inclusive in the final state Higgs bosons and no cuts of any kind are applied to them.

## Results

In this section we show numerical results for cross sections and differential distributions. Given that the QCD corrections have already been presented in Ref. [26, 27], the discussion focuses more on their combination with the EW ones which are presented here for the first time. Nonetheless, some distributions were not shown in Ref. [26] and are therefore discussed here in more details. In the following, the $\mathrm{NNLO \; QCD \times NLO \; EW}$ predictions are sometimes referred to as *state-of-the-art predictions*. Finally, the differences between the *full* and the *VBF* computation are highlighted.

**Table 1:** The fiducial cross section for the process $\text {p} \text {p} \rightarrow \text {H} \text {H} \text {j} \text {j}$, expressed in${\,\text {fb}}$ and in per cent, computed according to Eq. (4) at $14 \,\,\text {TeV}$ and under the selection cuts given in Sect. 2. The numbers in per cent are with respect to the LO cross section. The errors given in parenthesis are purely statistical whereas the additional uncertainties quoted for $\sigma ^\mathrm{full}_\mathrm{LO}$ and $\sigma _\mathrm{NNLO \; QCD \times NLO \; EW}$ are the QCD scale variations. We also show $\delta ^\mathrm{NF}_\mathrm{NNLO\; QCD}$ separately. The value of the correction factor to go from the VBF approximation to the full computation is $K_{\mathrm {full/VBF}}=0.99220(11)$

$\sigma ^\mathrm{full}_\mathrm{LO}$	$\delta ^\mathrm{full}_\mathrm{NLO\; QCD}$	$\delta ^\mathrm{VBF}_\mathrm{NNLO\; QCD}$	$\delta ^\mathrm{full}_\mathrm{NLO \; EW}$	$\sigma _\mathrm{NNLO \; QCD \times NLO \; EW}$	$\delta ^\mathrm{NF}_\mathrm{NNLO\; QCD}$ (fb)
$0.78444(9)^{+\,0.0825}_{-\,0.0694}$	$-\,0.07110(13)$	$-\,0.0115(5)$	$-\,0.0476(2)$	$0.6684(5)^{+\,0.002}_{-\,0.0004}$	0.01237(2)
$^{+\,10.5\%}_{-\,8.8\%}$	$-\,9.1 \%$	$-\,1.5 \%$	$-\,6.1 \%$	${-\,14.8 \%}^{+\,0.3\%}_{-\,0.06\%}$	$+\,1.7 \%$

In Table 1, fiducial cross sections and higher-order corrections are displayed for the event selection presented in Sect. 2. They are expressed both in femto barn and in per cent. The numbers in per cent are with respect to the LO cross section. The numbers in parenthesis indicate the statistical error while the additional information on $\sigma ^\mathrm{full}_\mathrm{LO}$ and $\sigma _\mathrm{NNLO \; QCD \times NLO \; EW}$ gives the scale variation estimate. Note that the total statistical uncertainty is not obtained by adding the individual statistical uncertainties in quadrature, as these are all correlated.

One of the main messages of Table 1 is that the QCD corrections are negative as for similar signatures such as single Higgs-production via VBF or VBS at the LHC. In addition, the higher-order QCD corrections dramatically reduce the uncertainty associated with missing QCD higher orders. In particular, it goes from $\left[ +\,10.5\% , -\,8.8\%\right]$ at LO to $\left[ {+\,0.3\%}, {-\,0.06\%}\right]$ at NNLO in QCD. We note that the non-factorisable NNLO QCD corrections are the only positive corrections, and that their contribution almost exactly cancels the factorisable NNLO QCD corrections. This is a coincidence of the particular cuts used here.

The second important point is the size of the EW corrections. It has recently been found (and further confirmed in Refs. [43, 64]) that large EW corrections are an intrinsic feature of VBS at the LHC [49]. It originates from the quantum numbers of the particles involved in the process as well as the large scale induced by the massive t-channel exchange [65]. For such processes, the corrections reach about $-\,15$ to $-\,20\%$ of the LO prediction. On the other hand, for single-Higgs production via VBF, EW corrections have been found to be around $-\,5\%$ [38, 46]. It is thus interesting to observe that, despite having a higher typical scale, the magnitude of the EW corrections for double-Higgs production via VBF is very close to the single-Higgs one. In particular, in VBS the typical scale (the invariant mass of the four leptons) is $\langle m_{4 \ell } \rangle \sim 390\,\,\text {GeV}$ while the VBF case it is even larger with $\langle m_{\text {H} \text {H}} \rangle \sim 610\,\,\text {GeV}$. In the same way as in Ref. [49], one can derive a leading-logarithmic approximation using Ref. [66]. Because the quantum numbers of the Higgs boson, such as the effective EW Casimir operator (see Eq. (B.10) in Ref. [66]), are significantly smaller than the ones of the Z or W gauge bosons, the logarithm coefficients are reduced with respect to the VBS case. For example, the coefficient of the double logarithms, which is directly proportional to the effective EW Casimir operator, is smaller by about a factor two. This implies, that VBF does not feature intrinsic large EW corrections as VBS.

The QCD corrections on the other hand tend to be somewhat smaller for double-Higgs production compared to single Higgs. This is due to the larger energy transfer in the t-channel which leads to harder jets and a higher dijet invariant mass. This in turn means that fewer events are lost due to QCD radiation. Overall, the state-of-the-art prediction displays a correction of about $-\,15\%$ with respect to the LO prediction. Finally, the numerical value of the correction factor is $K_{\mathrm{full/VBF}} = 0.99220(11)$ at the level of the fiducial cross section. It means that for the (rather exclusive) fiducial volume chosen here, the VBF approximation is reliable below the per-cent level. As shown later, this correction factor is not constant over the kinematic range and thus motivates its incorporation in our final predictions.

**Fig. 2:**
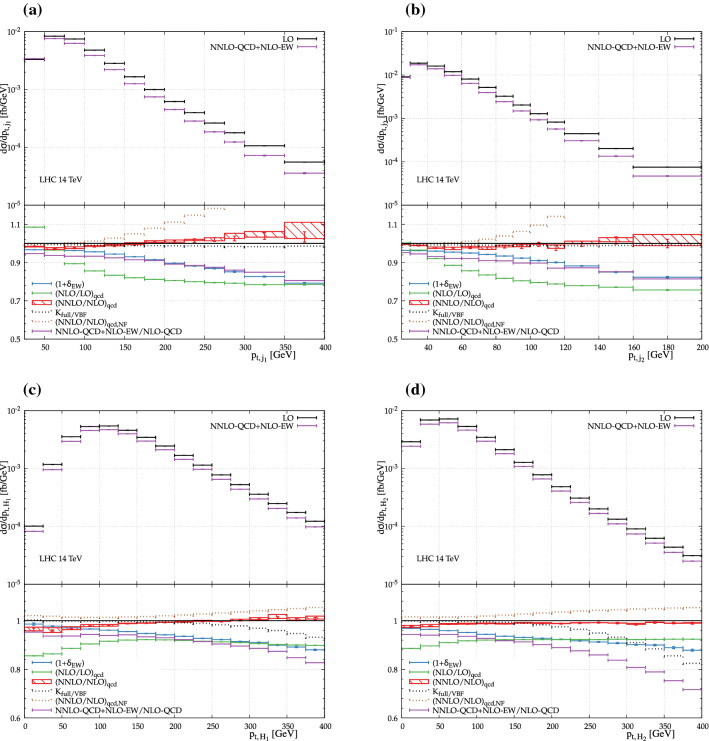
Differential distributions for $\text {p} \text {p} \rightarrow \text {j} \text {j} \text {H} \text {H}$ at the LHC with centre-of-mass energy of $14\,\,\text {TeV}$: **a** transverse momentum of the hardest jet (top left), **b** transverse momentum of the second hardest jet (top right), **c** transverse momentum of the hardest Higgs boson (bottom left), and **d** transverse momentum of the second hardest Higgs boson (bottom right). The upper panel shows the absolute contributions at NNLO QCD + NLO EW and the LO prediction. The lower panel shows the relative corrections. The bands denote the envelope of the QCD scale variation. Note that the non-factorisable corrections to the transverse momenta of the jets should not be trusted at large values, as the underlying eikonal approximation breaks down

In Fig. 2, several transverse-momentum distributions are shown. The first two are the transverse momentum of the hardest jet and second hardest jet. Comparing the EW corrections to the QCD ones, we observe that their shapes are rather similar. These corrections are driven by Sudakov logarithms that grow negatively large towards the high-energy region. In this case, high energy refers to the high transverse momenta. For the hardest jet, it goes to about $-\,25\%$ at $400\,\,\text {GeV}$, while for the second hardest jet, the value $-\,25\%$ is reached already at $200\,\,\text {GeV}$. Concerning the transverse momenta of the Higgs bosons, ordered by their transverse momentum, the overall behaviour is similar and the corrections grow smoothly towards higher momenta. It is interesting to notice that in this case the difference between the full and the VBF computation is of 10–20% at $400\,\,\text {GeV}$. This is the most striking effect of the VBF approximation that we have observed in the present study. But it happens in a rather suppressed part of the phase space. For other distributions, the results are very much in line with Ref. [55] where the difference between the full and the VBF computation have been found to be small.

**Fig. 3:**
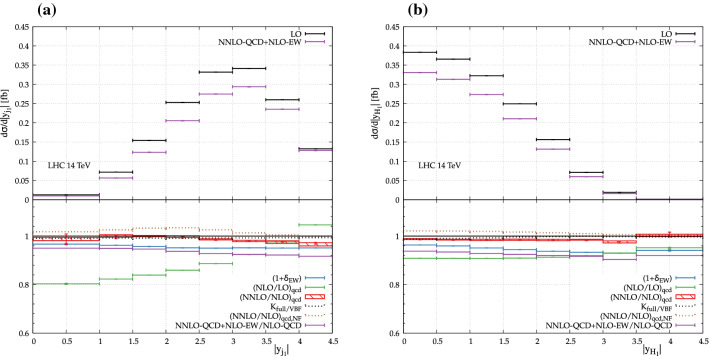
Differential distributions for $\text {p} \text {p} \rightarrow \text {j} \text {j} \text {H} \text {H}$ at the LHC with centre-of-mass energy of $14\,\,\text {TeV}$: **a** rapidity of the hardest jet (left) and **b** rapidity of the hardest Higgs boson (right). The upper panel shows the absolute contributions at NNLO QCD + NLO EW and the LO prediction. The lower panel shows the relative corrections. The bands denote the envelope of the QCD scale variation

The rapidity distributions of the hardest jet and Higgs boson are shown in Fig. 3. For the rapidity distribution of the hardest jet, the NLO QCD corrections go from $-\,20\%$ at 0 rapidity to about $+\,4\%$ at 4.5 rapidity. The NNLO QCD corrections are smaller and vary within $4\%$ across the phase space. The NLO EW corrections, on the other hand, hardly change over the displayed rapidity range and essentially inherit the overall normalisation. The rapidity distribution of the hardest Higgs boson displays even more stable corrections. The NLO QCD corrections fluctuate by less than $5\%$ over the whole spectrum and the NNLO QCD corrections are also very stable (few per cent variation). The NLO EW corrections show a small shape distortion at the per-cent level. In both cases, the difference between the full and the VBF computation is minimal and do not exceed few per cent. Note also that for these distributions, the NNLO non-factorisable corrections are rather suppressed with small variations over the kinematic range shown.

**Fig. 4:**
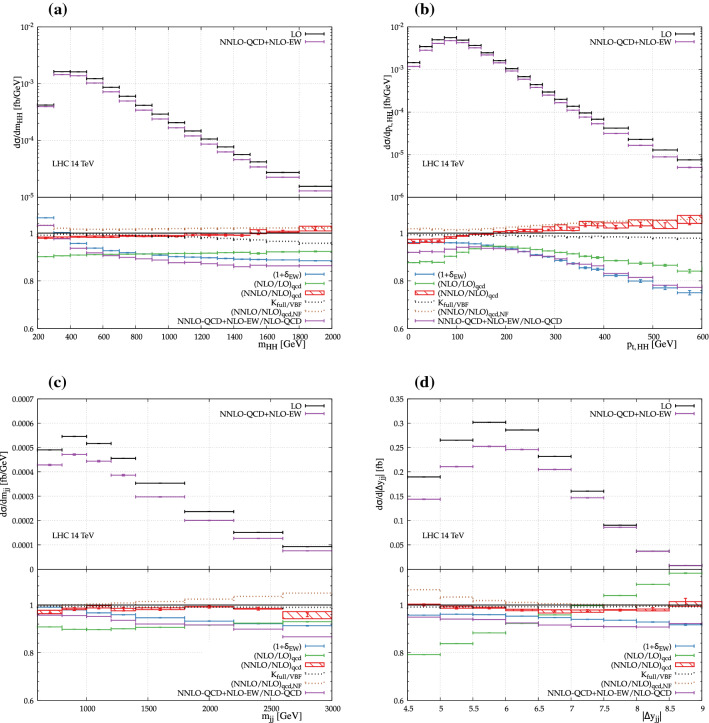
Differential distributions for $\text {p} \text {p} \rightarrow \text {j} \text {j} \text {H} \text {H}$ at the LHC with centre-of-mass energy of $14\,\,\text {TeV}$: **a** invariant mass of the two Higgs bosons (top left), **b** transverse momentum of the two Higgs bosons (top right), **c** invariant mass of the two hardest jets (bottom left), and **d** rapidity difference between the two hardest jets (bottom right). The upper panel shows the absolute contributions at NNLO QCD + NLO EW and the LO prediction. The lower panel shows the relative corrections. The bands denote the envelope of the QCD scale variation

Finally, in Fig. 4 the invariant-mass and transverse-momentum distributions of the di-Higgs system are shown. For the invariant mass, the EW corrections are positive (above $5\%$) at $200\,\,\text {GeV}$. In the second and third bin, the maximum of the distribution is reached. The corrections become then negative about $-\,10\%$ at $2\,\,\text {TeV}$. The QCD corrections on the other hand are rather flat and largely inherit the overall normalisation. In the tail of the distribution at about $2\,\,\text {TeV}$, the VBF computation start to diverge from the full one at a level of $5\%$. The transverse momentum of the two Higgs bosons displays much larger shape distortions. The NLO QCD corrections are maximal at low and high transverse momentum and are minimal around $200\,\,\text {GeV}$. This originates from kinematic constraints at low transverse momentum i.e. in the first three bins (the cut on the jet transverse momenta being $25\,\,\text {GeV}$). With higher-order QCD radiation, this constraint is relaxed allowing a relative increase in the cross section. At high transverse momentum, the running of the strong coupling is then taking place. Such an effect has already been observed for single Higgs production [32] and is also visible (even if less pronounced) in the transverse-momentum distributions of the Higgs boson in Fig. 2c and d. The NNLO QCD corrections slowly increase towards high transverse momentum to $+\,5\%$ at $600\,\,\text {GeV}$. The EW corrections on the other hand displays a typical Sudakov behaviour. The corrections become rather large ($\simeq -25\%$) at $600\,\,\text {GeV}$. But such an energy corresponds to a rather extreme part of the phase space which is suppressed by more than three orders of magnitude with respect to the maximum of the distribution. Finally, the invariant mass and the rapidity difference of the two tagging jets are also displayed. These are typical observables used by experimental collaborations to enhance the VBF signal. Going to high invariant mass, the EW corrections slightly increase to reach about $-\,10\%$. This value is also obtained for larger rapidity difference of the two tagging jets. Such a kinematic (large invariant mass and large rapidity difference) is the typical VBF kinematic meaning that making the phase space cuts even more exclusive would increase the EW corrections but not dramatically. Overall, both at the level of the cross section and for differential distribution, the findings regarding the EW corrections are very similar to the ones for single Higgs production [38].

Finally, a few remarks regarding the non-factorisable NNLO QCD corrections. For most observable shown here the non-factorisable NNLO QCD corrections are of roughly the same size as the factorisable NNLO QCD corrections, although shapes differ for most observables. This warrants their inclusion when high-precision predictions are necessary. However, one has to be careful as the non-factorisable corrections are here computed in the eikonal approximation, which is only valid whenever all transverse momentum scales are much smaller than the partonic centre-of-mass energy. In particular this means that the approximation breaks down whenever the transverse momentum of a jet becomes large. As can be seen in Fig. 2a, b in this region the non-factorisable NNLO QCD corrections can grow very large, and they should no longer be trusted. For all other observables shown here the eikonal approximation is expected to be valid as discussed in Ref. [27].

## Conclusion

The high-luminosity phase of the LHC will allow to probe rare processes, giving insight into fundamental interactions at higher energies. One of these rare processes is the production of two Higgs bosons through VBF. To that end, SM predictions are critical for the measurements of the process and associated searches for new physics phenomena.

In the present article we have provided state-of-the-art predictions within the SM in a realistic experimental set-up at the LHC at $14\,\,\text {TeV}$. They feature the full LO predictions wit NLO QCD and EW corrections without relying on any approximation. These have then been combined with the existing NNLO QCD corrections to obtain the first predictions at $\mathrm{NNLO \; QCD \times NLO \; EW}$. The NLO EW corrections are presented for the first time here as well as the corresponding state-of-the-art predictions. Also, some of the differential distributions are shown at NNLO QCD here for the first time.

We have found that the EW corrections display the typical Sudakov behaviour in the high-energy limits. The corrections are of $-\,6\%$ for the fiducial cross section while they can typically grow up to $-\,10$ to $-\,20\%$ in differential distributions. In general, the corrections are rather similar to the ones for single-Higgs productions and do not display very large EW corrections. The situation is thus rather close to the one of QCD corrections that largely share similarities between single- and double-Higgs production.

For the event selection chosen here, we predict a fiducial cross section of$0.67{\,\text {fb}}$. This amounts to a correction factor of $-\,14.8\%$ with respect to the LO predictions. At the level of the differential distributions, the corrections can be larger and give rise to shape distortions of the order of $40\%/50\%$. We note that for the High Luminosity LHC with an expected integrated luminosity of$3000{\,\text {fb}} ^{-1}$ this amounts to more than 2000 events in the rather strict fiducial volume used here.

Finally, we have also analysed the differences between the full computation including *s*-channel contributions and the VBF approximated one. This is also the first time that such results are shown in that respect. They are in rather good agreement with previous findings for single-Higgs production. In line with previous studies for similar processes, we have found that the VBF approximation becomes unreliable in rather inclusive set-ups or in extreme regions of the phase space.

The present results provide detailed information regarding higher-order corrections in double-Higgs production via VBF. It should thus be used as a guideline by the experimental collaborations in their quests for the measurement of this process during the high-luminosity phase of the LHC. It can also serve as a reference in the corresponding new-physics searches for future collider experiments that will help us unravel the mysteries of fundamental physics.

## Data Availability

This manuscript has no associated data or the data will not be deposited. [Authors’ comment: Predictions derived from the calculation presented in this article can nonetheless be obtained from the authors upon request].

## Footnotes

1 Using the same phase-space cuts, we have recomputed the photon-induced corrections with HAWK [44] for single Higgs production via VBF using the NNPDF31_nnlo_as_0118_luxqed set [45]. We have found that this contribution amounts to $0.9\%$ of the fiducial cross section.

2 For the NNLO corrections the width has been used for the internal Higgs propagators.

## References

[1] ATLAS Collaboration, G. Aad, et al., Observation of a new particle in the search for the Standard Model Higgs boson with the ATLAS detector at the LHC. Phys. Lett. B **716**, 1–29 (2012). 10.1016/j.physletb.2012.08.020. arXiv:1207.7214 [hep-ex]

[2] CMS Collaboration, S. Chatrchyan et al., Observation of a new boson at a mass of 125 GeV with the CMS experiment at the LHC. Phys. Lett. B **716**, 30–61 (2012). 10.1016/j.physletb.2012.08.021. arXiv:1207.7235 [hep-ex]

[3] P. Azzi et al., Report from Working Group 1. CERN Yellow Rep. Monogr. **7**, 1–220 (2019). 10.23731/CYRM-2019-007.1. arXiv:1902.04070 [hep-ph]

[4] ATLAS Collaboration, M. Aaboud, et al., A search for resonant and non-resonant Higgs boson pair production in the $${b\bar{b}\tau ^+\tau ^-}$$ decay channel in $$pp$$ collisions at $$\sqrt{s}=13$$ TeV with the ATLAS detector. Phys. Rev. Lett. (2018). arXiv:1808.00336 [hep-ex] 10.1103/PhysRevLett.121.191801 30468613

[5] ATLAS Collaboration, M. Aaboud, et al., Search for Higgs boson pair production in the $$\gamma \gamma WW^{*}$$ channel using $$pp$$ collision data recorded at $$\sqrt{s} = 13$$ TeV with the ATLAS detector. Eur. Phys. J. (2018). arXiv:1807.08567 [hep-ex] 10.1140/epjc/s10052-018-6457-x PMC6383910 30872957

[6] ATLAS Collaboration, M. Aaboud, et al., Search for Higgs boson pair production in the $$\gamma \gamma b\bar{b}$$ final state with 13 TeV $$pp$$ collision data collected by the ATLAS experiment. arXiv:1807.04873 [hep-ex]

[7] ATLAS Collaboration, M. Aaboud, et al., Search for pair production of Higgs bosons in the $$b\bar{b}b\bar{b}$$ final state using proton–proton collisions at $$\sqrt{s} = 13$$ TeV with the ATLAS detector. arXiv:1804.06174 [hep-ex]

[8] ATLAS Collaboration, M. Aaboud, et al., Search for pair production of Higgs bosons in the $$b\bar{b}b\bar{b}$$ final state using proton–proton collisions at $$\sqrt{s} = 13$$ TeV with the ATLAS detector. Phys. Rev. D **94**(5), 052002 (2016). 10.1103/PhysRevD.94.052002. arXiv:1606.04782 [hep-ex]

[9] ATLAS Collaboration, G. Aad, et al., Searches for Higgs boson pair production in the $$hh\rightarrow bb\tau \tau , \gamma \gamma WW^*, \gamma \gamma bb, bbbb$$ channels with the ATLAS detector. Phys. Rev. D **92**, 092004 (2015). 10.1103/PhysRevD.92.092004. arXiv:1509.04670 [hep-ex]

[10] ATLAS Collaboration, G. Aad, et al., Search for Higgs boson pair production in the $$b\bar{b}b\bar{b}$$ final state from pp collisions at $$\sqrt{s} = 8$$ TeV with the ATLAS detector. Eur. Phys. J. C **75**(9), 412 (2015). 10.1140/epjc/s10052-015-3628-x. arXiv:1506.00285 [hep-ex] 10.1140/epjc/s10052-015-3628-x PMC4564859 26380565

[11] ATLAS Collaboration, G. Aad, et al., Search For Higgs Boson Pair Production in the $$\gamma \gamma b\bar{b}$$ final state using $$pp$$ collision data at $$\sqrt{s}=8$$ TeV from the ATLAS detector. Phys. Rev. Lett. **114**(8), 081802 (2015). 10.1103/PhysRevLett.114.081802. arXiv:1406.5053 [hep-ex] 10.1103/PhysRevLett.114.081802 25768755

[12] CMS Collaboration, A.M. Sirunyan, et al., Search for nonresonant Higgs boson pair production in the $$\rm b\overline{b}b\overline{b}$$ final state at $$\sqrt{s} =$$ 13 TeV. arXiv:1810.11854 [hep-ex]

[13] CMS Collaboration, A.M. Sirunyan, et al., Search for Higgs boson pair production in the $$\gamma \gamma \rm b\overline{b}$$ final state in pp collisions at $$\sqrt{s}=$$ 13 TeV. arXiv:1806.00408 [hep-ex]

[14] CMS Collaboration, A.M. Sirunyan et al., Search for resonant and nonresonant Higgs boson pair production in the $$ \rm b\overline{\rm b}\rm \rm \ell \nu \ell \nu \rm $$ final state in proton-proton collisions at $$ \sqrt{s}=13 $$ TeV. JHEP **01**, 054 (2018). 10.1007/JHEP01(2018)054. arXiv:1708.04188 [hep-ex]

[15] CMS Collaboration, A.M. Sirunyan et al., Search for Higgs boson pair production in events with two bottom quarks and two tau leptons in proton–proton collisions at $$\sqrt{s}$$ =13 TeV. Phys. Lett. B **778**, 101–127 (2018). 10.1016/j.physletb.2018.01.001. arXiv:1707.02909 [hep-ex]

[16] CMS Collaboration, A.M. Sirunyan et al., Search for Higgs boson pair production in the $$bb\tau \tau $$ final state in proton–proton collisions at $$\sqrt{(}s)=8 \rm $$ TeV. Phys. Rev. D **96**(7), 072004 (2017). 10.1103/PhysRevD.96.072004. arXiv:1707.00350 [hep-ex]

[17] Baglio J, Djouadi A, Gröber R, Mühlleitner MM, Quevillon J, Spira M (2013). The measurement of the Higgs self-coupling at the LHC: theoretical status. JHEP.

[18] LHC Higgs Cross Section Working Group Collaboration, D. de Florian, et al., Handbook of LHC Higgs cross sections: 4. Deciphering the nature of the Higgs sector. arXiv:1610.07922 [hep-ph]

[19] de Florian D, Mazzitelli J (2013). Higgs Boson pair production at next-to-next-to-leading order in QCD. Phys. Rev. Lett..

[20] de Florian D, Grazzini M, Hanga C, Kallweit S, Lindert JM, Maierhöfer P, Mazzitelli J, Rathlev D (2016). Differential Higgs boson pair production at next-to-next-to-leading order in QCD. JHEP.

[21] de Florian D, Mazzitelli J (2015). Higgs pair production at next-to-next-to-leading logarithmic accuracy at the LHC. JHEP.

[22] Grazzini M, Heinrich G, Jones S, Kallweit S, Kerner M, Lindert JM, Mazzitelli J (2018). Higgs boson pair production at NNLO with top quark mass effects. JHEP.

[23] Bishara F, Contino R, Rojo J (2017). Higgs pair production in vector-boson fusion at the LHC and beyond. Eur. Phys. J. C.

[24] ATLAS, CMS Collaboration, G. Aad, et al., Measurements of the Higgs boson production and decay rates and constraints on its couplings from a combined ATLAS and CMS analysis of the LHC pp collision data at $$ \sqrt{s}=7 $$ and 8 TeV. JHEP **08**, 045 (2016). 10.1007/JHEP08(2016)045. arXiv:1606.02266 [hep-ex]

[25] ATLAS Collaboration, G. Aad, et al., Search for the $$HH \rightarrow b \bar{b} b \bar{b}$$ process via vector-boson fusion production using proton–proton collisions at $$\sqrt{s} = 13$$ TeV with the ATLAS detector. arXiv:2001.05178 [hep-ex]

[26] Dreyer FA, Karlberg A (2019). Fully differential vector-boson fusion Higgs pair production at next-to-next-to-leading order. Phys. Rev. D.

[27] F.A. Dreyer, A. Karlberg, L. Tancredi, On the impact of non-factorisable corrections in VBF single and double Higgs production. arXiv:2005.11334 [hep-ph]

[28] Dreyer FA, Karlberg A (2018). Vector-boson fusion Higgs pair production at N$$^3$$LO. Phys. Rev. D.

[29] Figy T (2008). Next-to-leading order QCD corrections to light Higgs pair production via vector boson fusion. Mod. Phys. Lett. A.

[30] Bolzoni P, Maltoni F, Moch S-O, Zaro M (2010). Higgs production via vector-boson fusion at NNLO in QCD. Phys. Rev. Lett..

[31] Ling L-S, Zhang R-Y, Ma W-G, Guo L, Li W-H, Li X-Z (2014). NNLO QCD corrections to Higgs pair production via vector boson fusion at hadron colliders. Phys. Rev. D.

[32] M. Cacciari, F.A. Dreyer, A. Karlberg, G.P. Salam, G. Zanderighi, Fully differential vector-boson-fusion Higgs production at next-to-next-to-leading order. Phys. Rev. Lett. **115**(8), 082002 (2015). 10.1103/PhysRevLett.115.082002. 10.1103/PhysRevLett.120.139901. arXiv:1506.02660 [hep-ph] (**Erratum: Phys. Rev. Lett. 120, no. 13, 139901(2018)**) 10.1103/PhysRevLett.120.139901 29694213

[33] Dreyer FA, Karlberg A (2016). Vector-boson fusion Higgs production at three loops in QCD. Phys. Rev. Lett..

[34] Cruz-Martinez J, Gehrmann T, Glover EWN, Huss A (2018). Second-order QCD effects in Higgs boson production through vector boson fusion. Phys. Lett. B.

[35] Frederix R, Frixione S, Hirschi V, Maltoni F, Mattelaer O, Torrielli P, Vryonidou E, Zaro M (2014). Higgs pair production at the LHC with NLO and parton-shower effects. Phys. Lett. B.

[36] Ballestrero A (2018). Precise predictions for same-sign W-boson scattering at the LHC. Eur. Phys. J. C.

[37] Campanario F, Figy TM, Plätzer S, Rauch M, Schichtel P, Sjödahl M (2018). Stress testing the vector-boson-fusion approximation in multijet final states. Phys. Rev. D.

[38] Ciccolini M, Denner A, Dittmaier S (2008). Electroweak and QCD corrections to Higgs production via vector-boson fusion at the LHC. Phys. Rev. D.

[39] Harlander RV, Vollinga J, Weber MM (2008). Gluon-induced weak boson fusion. Phys. Rev. D.

[40] Bolzoni P, Maltoni F, Moch S-O, Zaro M (2012). Vector boson fusion at NNLO in QCD: SM Higgs and beyond. Phys. Rev. D.

[41] Liu T, Melnikov K, Penin AA (2019). Nonfactorizable QCD effects in Higgs boson production via vector boson fusion. Phys. Rev. Lett..

[42] Biedermann B, Denner A, Pellen M (2017). Complete NLO corrections to W$$^{+}$$W$$^{+}$$ scattering and its irreducible background at the LHC. JHEP.

[43] Denner A, Dittmaier S, Maierhöfer P, Pellen M, Schwan C (2019). QCD and electroweak corrections to WZ scattering at the LHC. JHEP.

[44] Denner A, Dittmaier S, Kallweit S, Mück A (2015). HAWK 2.0: a Monte Carlo program for Higgs production in vector-boson fusion and Higgs strahlung at hadron colliders. Comput. Phys. Commun..

[45] NNPDF Collaboration, V. Bertone, S. Carrazza, N.P. Hartland, J. Rojo, Illuminating the photon content of the proton within a global PDF analysis. Sci. Post Phys. **5**(1), 008 (2018). 10.21468/SciPostPhys.5.1.008. arXiv:1712.07053 [hep-ph]

[46] Ciccolini M, Denner A, Dittmaier S (2007). Strong and electroweak corrections to the production of Higgs + 2jets via weak interactions at the LHC. Phys. Rev. Lett..

[47] Figy T, Palmer S, Weiglein G (2012). Higgs production via weak boson fusion in the standard model and the MSSM. JHEP.

[48] J. Baglio, et al., Release Note-VBFNLO 2.7.0. arXiv:1404.3940 [hep-ph]

[49] Biedermann B, Denner A, Pellen M (2017). Large electroweak corrections to vector-boson scattering at the large hadron collider. Phys. Rev. Lett..

[50] S. Actis, A. Denner, L. Hofer, J.-N. Lang, A. Scharf, S. Uccirati, RECOLA: REcursive computation of one-loop amplitudes. arXiv:1605.01090 [hep-ph]

[51] Actis S, Denner A, Hofer L, Scharf A, Uccirati S (2013). Recursive generation of one-loop amplitudes in the standard model. JHEP.

[52] Denner A, Lang J-N, Uccirati S (2018). Recola2: REcursive computation of one-loop amplitudes 2. Comput. Phys. Commun..

[53] Denner A, Dittmaier S, Hofer L (2014). Collier-a fortran-library for one-loop integrals. PoS LL.

[54] Denner A, Dittmaier S, Hofer L (2017). Collier: a fortran-based complex one-loop LIbrary in extended regularizations. Comput. Phys. Commun..

[55] LHC Higgs Cross Section Working Group Collaboration, J.R. Andersen, et al., Handbook of LHC Higgs cross sections: 3. Higgs properties. arXiv:1307.1347 [hep-ph]

[56] Buckley A, Ferrando J, Lloyd S, Nordström K, Page B, Rüfenacht M, Schönherr M, Watt G (2015). LHAPDF6: parton density access in the LHC precision era. Eur. Phys. J. C.

[57] Bardin DY, Leike A, Riemann T, Sachwitz M (1988). Energy-dependent width effects in $${e}^+ {e}^-$$-annihilation near the Z-boson pole. Phys. Lett. B.

[58] Denner A, Dittmaier S, Roth M, Wackeroth D (2000). Electroweak radiative corrections to $${e}^+ {e}^- \rightarrow W W \rightarrow $$ 4 fermions in double pole approximation: the RACOONWW approach. Nucl. Phys. B.

[59] Denner A, Dittmaier S, Roth M, Wackeroth D (1999). Predictions for all processes $${e}^+ {e}^- \rightarrow $$ 4 fermions $$+ \gamma $$. Nucl. Phys. B.

[60] A. Denner, S. Dittmaier, M. Roth, L.H. Wieders, Electroweak corrections to charged-current $${e}^+ {e}^- \rightarrow $$ 4 fermion processes: technical details and further results. Nucl. Phys. B **724**, 247–294 (2005). 10.1016/j.nuclphysb.2011.09.001, 10.1016/j.nuclphysb.2005.06.033. arXiv:hep-ph/0505042 504) (**Erratum: Nucl. Phys. B 854 (2012)**)

[61] Denner A, Dittmaier S (2006). The complex-mass scheme for perturbative calculations with unstable particles. Nucl. Phys. Proc. Suppl..

[62] Cacciari M, Salam GP, Soyez G (2008). The anti-$$k_t$$ jet clustering algorithm. JHEP.

[63] Cacciari M, Salam GP, Soyez G (2012). Fastjet user manual. Eur. Phys. J. C.

[64] Chiesa M, Denner A, Lang J-N, Pellen M (2019). An event generator for same-sign W-boson scattering at the LHC including electroweak corrections. Eur. Phys. J. C.

[65] Denner A, Hahn T (1998). Radiative corrections to $${\rm W}^+ {\rm W}^- \rightarrow {\rm W}^+ {\rm W}^-$$ in the electroweak standard model. Nucl. Phys. B.

[66] Denner A, Pozzorini S (2001). One loop leading logarithms in electroweak radiative corrections. 1. Results. Eur. Phys. J. C.

